# Four anastomotic techniques following transanal total mesorectal excision (TaTME)

**DOI:** 10.1007/s10151-015-1414-2

**Published:** 2016-01-12

**Authors:** M. Penna, J. J. Knol, J. B. Tuynman, P. P. Tekkis, N. J. Mortensen, R. Hompes

**Affiliations:** Department of Colorectal Surgery, Churchill Hospital, University Hospitals of Oxford, Old Road, Oxford, OX3 7LE UK; Department of Colorectal Surgery, Jessa Hospital, Hasselt, Belgium; Department of Surgery, VU University Medical Centre, Amsterdam, The Netherlands; Department of Colorectal Surgery, Royal Marsden Hospital, London, UK

**Keywords:** Transanal, TME, Bottom-up, Anastomosis, Laparoscopy, Outcomes

## Abstract

Transanal total mesorectal excision (TaTME) is a novel approach pioneered to tackle the challenges posed by difficult pelvic dissections in rectal cancer and the restrictions in angulation of currently available laparoscopic staplers. To date, four techniques can be employed in order to create the colorectal/coloanal anastomosis following TaTME. We present a technical note describing these techniques and discuss the risks and benefits of each.

**Electronic supplementary material:**

The online version of this article (doi:10.1007/s10151-015-1414-2) contains supplementary material, which is available to authorized users.

## Introduction

Transanal total mesorectal excision (TaTME) is a novel approach that has emerged following technical advances in minimally invasive surgery [1], transanal endoscopic microsurgery (TEM) [2], and natural orifice transluminal approaches [3].

After the combined laparoscopic and transanal TME dissection, specimen removal and formation of an anastomosis are critical steps of the TaTME procedure. In addition to hand-sewn coloanal anastomosis, three stapling techniques for the colorectal anastomosis have been employed: a stapled anastomosis using the EEA™ Haemorrhoid Stapler (AutoSuture; Covidien, Dublin, Ireland) [4], a standard diameter circular stapler either in combination with a guiding 10Fr redivac drain [5] or a pull-through method. In this technical note, we describe the different anastomotic techniques in detail and discuss their main differences.

## Technical note

### Traditional hand-sewn coloanal anastomosis

**Fig. 1:**
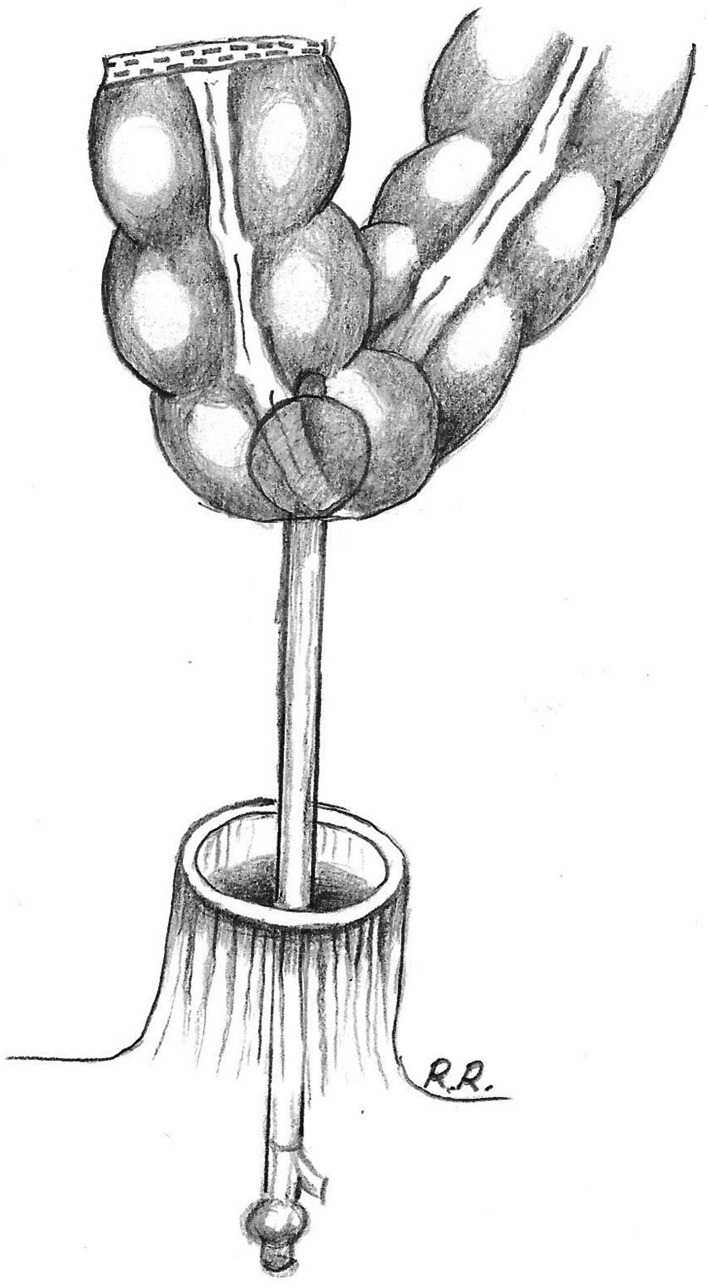
In preparation for a hand-sewn anastomosis, a 14Fr Foley catheter inserted into the lumen of the bowel can help deliver the colonic conduit into the anal canal avoiding any twist

The descending colon is delivered into the pelvis and brought into position for a coloanal hand-sewn anastomosis. A 14Fr Foley catheter inserted into the lumen can be useful to help deliver the colonic conduit into the anal canal avoiding any twist (Fig. 1). Alternatively, tagging sutures can be placed into the proximal colon to guide the colonic conduit down. A self-retaining retractor is positioned to improve exposure and obtain adequate views of the anorectal stump wall. Commonly used retractors are the Lone Star (Lone Star Medical Products Inc., Houston, TX, USA) or the Scott Ring retractors (Lone Star Medical Products, Stafford, TX, USA). A one-layer (or two-layer) anastomosis is then fashioned using interrupted polyglycolic acid 2/0 or 3/0 sutures, as originally described by Sir Alan Parks [6]. Each suture incorporates the mucosa of the anorectal cuff, a portion of the upper internal sphincter and full-thickness muscular layer of the colon. The anastomosis can be constructed as a side-to-end anastomosis, colonic J-pouch, or straight (end-to-end) anastomosis.

### Double pursestring circular stapled anastomosis: three techniques

If oncologically safe, it is advised to perform a stapled colorectal anastomosis, which tends to result in better functional outcome due to higher length of the rectal cuff. Compared to standard laparoscopic or open stapling of the distal rectum, the TaTME allows stapling techniques with excellent visualisation and avoidance of cross stapling, especially in a male patient with narrow pelvis and obese patients. As a result, the TaTME procedure may lead to lower leakage rates and better functional and oncological outcomes. However, more data from large international cohorts and randomised trials are awaited.

The main difference for a stapled intestinal reconstruction compared to a standard laparoscopic anterior resection is the open rectal stump after a TaTME procedure. A key aspect to ensure a reliable anastomosis is a full-thickness pursestring suture (monofilament polypropylene suture 2/0) of the open rectal stump. Gaps in the pursestring need to be avoided as this can lead to defects in the anastomosis. Furthermore, it is important to ensure that only the anorectal wall is incorporated into the pursestring. Particularly in female patients, the surgeon has to carefully inspect the vaginal wall. The pursestring can be placed either through the access channel of the GelPoint Path (Applied Medical) for a colorectal anastomosis or within the anal canal for a coloanal anastomosis. A circular anal dilator can enhance exposure when dealing with a very low rectal cuff, which tends to retract into the anal canal [7]. After completing the pursestring, three different stapling techniques can be applied, each with its own advantage points, described below. As the anastomosis is close to the anal margin, it can be inspected after construction and reinforced if required under direct vision with hand placed interrupted sutures. The abdominal CO2 allows easy visualisation transanally of any air leak through the anastomosis. Similar to hand-sewn anastomoses, a side-to-end, colonic J-pouch or straight (end-to-end) anastomosis can be constructed.

#### EEA™ haemorrhoid stapled anastomosis

**Fig. 2:**
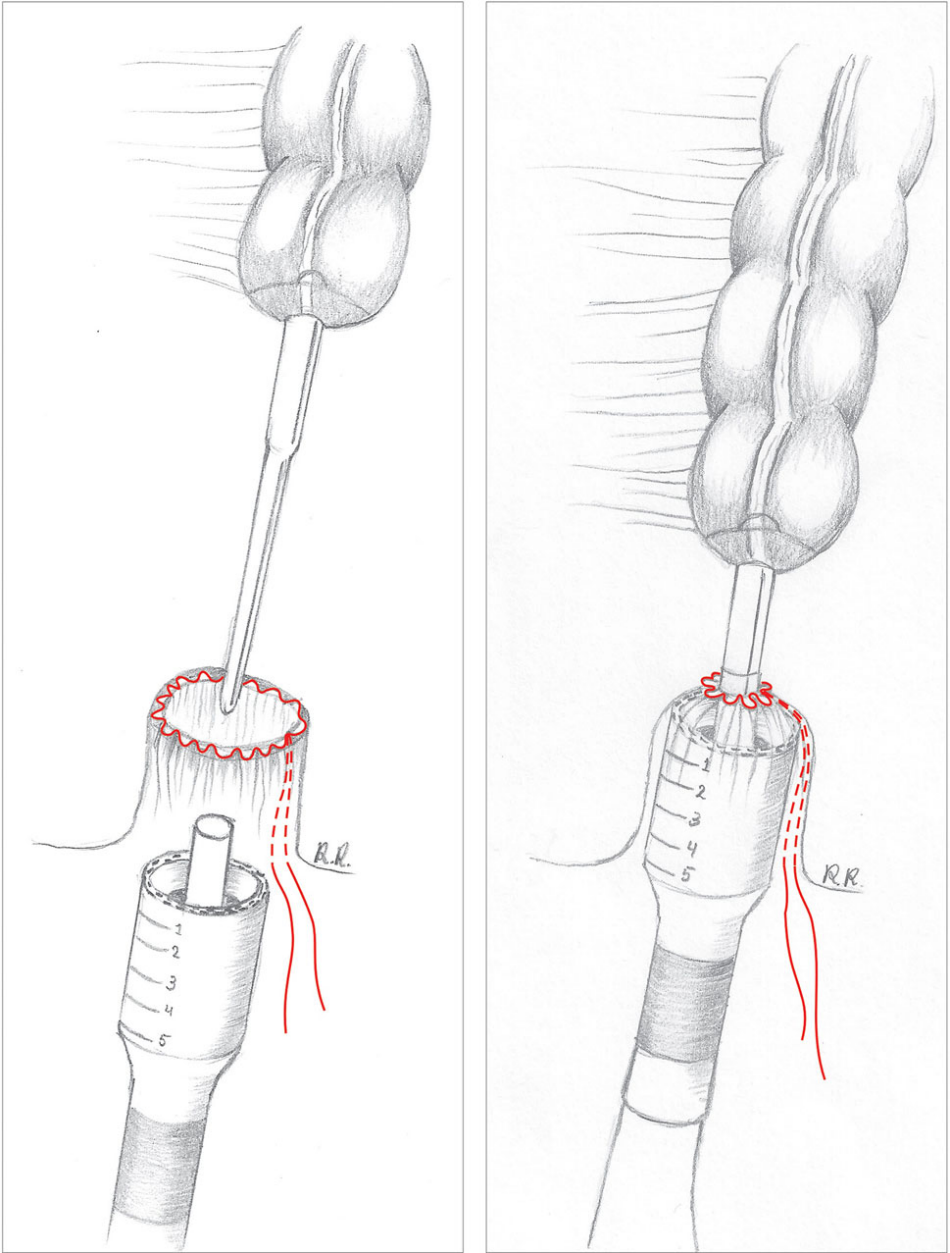
Pursestring is placed on the open anorectal stump, and the long spindle of the circular EEA™ stapler is brought transanally through the centre of the pursestring suture (*left image*). The anvil is connected to the centre shaft of the stapler, and the pursestring is then tightened around the centre rod (*right image*)

The proximal colon is prepared by inserting the detachable 33-mm circular stapling anvil (AutoSuture EEA™ haemorrhoid and prolapse DST series; Covidien) and securing a pursestring around the centre rod. Placement of a pursestring on the open anorectal stump then occurs. The extended reach of the centre rod on the anvil (13.5 cm) allows for sufficient access to pass it through the anal canal to connect with the stapler device before tying the rectal pursestring in a safe and efficient manner under direct vision (Fig. 2 and Video). The stapler is then closed, holding it perpendicular to the opening of the anus.

#### Modified circular stapled anastomosis 28–31 mm with abdominal view

**Fig. 3:**
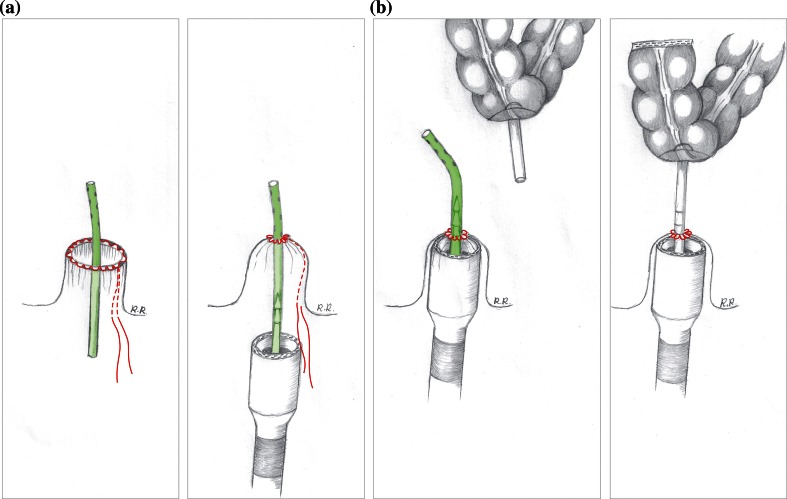
A 10Fr redivac drain is inserted through the central opening of the pursestring and secured by tying the pursestring (**a**). The spindle of a standard 28- or 31-mm AutoSuture CEEA™ circular stapler is attached to the distal end of the drain (**a**) and advanced into the pelvis (**b**). With the assistance of the laparoscopic graspers, the drain is removed, and the anvil is connected to the spindle ready to form the anastomosis (**b**)

A more recently described technique involves the use of a standard circular stapler and a 10Fr redivac drain [5, 7]. First, the proximal colon is prepared by securing the anvil of a standard 28- or 31-mm AutoSuture CEEA™ (Covidien) gun into the bowel. Then, a 10Fr redivac drain is inserted through the central opening of the pursestring into the pelvis and held in place by tying the rectal pursestring (Fig. 3a). The spindle of a standard 28- or 31-mm AutoSuture CEEA™ circular stapler is attached to the distal end of the drain and advanced into the pelvis (Fig. 3b). The redivac drain acts as a guide to ensure a perfect central position of the spindle through the centre of the pursestring. The laparoscopic operator is then able to remove the drain, uncovering the spindle intra-abdominally. With the assistance of the laparoscopic graspers, the anvil and spindle are connected, and the anastomosis is performed under direct laparoscopic vision (Fig. 3b).

#### Modified pull-through circular stapled anastomosis 28–31 mm with transanal view

**Fig. 4:**
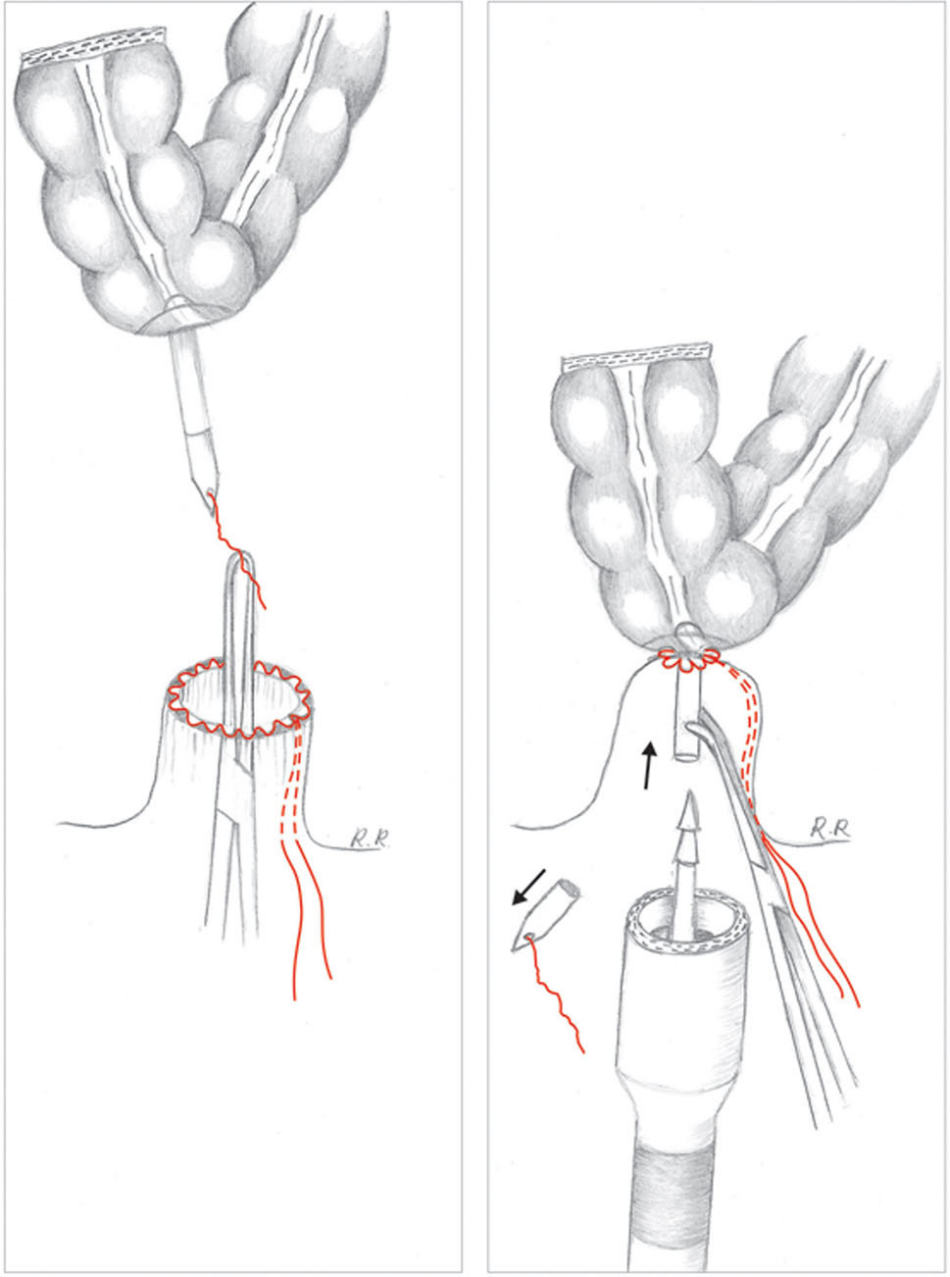
A multifilament suture is attached to the *white* plastic cap that is connected to the anvil which has been secured with a pursestring in the bowel. A laparoscopic grasper passed transanally grasps the multifilament suture and guides the anvil down to the rectal opening in order to tighten the second pursestring around the anvil. Whilst the anvil is held in place with a curved Roberts artery forceps, the *white cap* is removed, and the stapling gun attached allowing the anastomosis to be performed under direct vision

A novel technique involves the use of a standard circular stapler. The colon with the anvil is brought down to the pelvic floor using a 2.0-multifilament suture. First, the proximal colon is prepared with the anvil of the 28–31 mm circular stapling device in a conventional way. The supplied white plastic cap with attached a long multifilament suture is connected to the anvil. The proximal colon with the anvil is gently pulled down to the pelvic floor by grasping the multifilament suture attached to the anvil with a laparoscopic grasper inserted transanally. The anvil is brought through the anorectal stump opening so that the pursestring of the rectal stump can be tightened around the anvil enabling a tight and secure pursestring. Optimal exposure with the Lone Star retractor is essential. Whilst the anvil is held in place with a curved Roberts artery forceps, the white cap is removed and the stapling gun attached allows the anastomosis to be performed under direct vision (Fig. 4 and Video).

## Discussion

The formation of a colorectal or coloanal anastomosis is one of the critical steps post-TaTME that has been performed using both hand-sewn and stapling techniques. During a TaTME procedure, the distal rectal wall is divided at the start of the transanal dissection. This leaves an open distal rectal stump, which can easily be retracted and held in position for a hand-sewn anastomosis. The hand-sewn approach appears to be more suitable for very low coloanal anastomoses, as a pursestring closure is unlikely to be possible due to insufficient stump length. The level of the pursestring is dependent on the height of the tumour. If oncologically safe with an adequate margin, a rectal cuff just above the internal sphincter is preferred in order to have better functional outcome compared to the coloanal anastomosis. Conversely, a longer rectal stump may owe itself more readily to a stapling technique, as the visual exposure may be inadequate, and the distance from the anus too far for a hand-sewn anastomosis.

The EEA™ Haemorrhoid Stapler (Covidien) has been frequently used post-TaTME. The advantage of this stapler is the longer central rod on the anvil (13.5 cm) that allows connection to the stapler device before closure of the pursestring. However, there are two potential disadvantages associated with its use. The first is that the stapler’s large diameter of 33 mm could risk incorporating sphincter muscle or even the vagina into the stapler when forming a low coloanal anastomosis. This may lead to a worse functional outcome. Secondly, it is not always possible to fit the large-sized anvil into the new colonic conduit, even in a side-to-end orientation.

More recently, a stapling technique using the CEEA™ stapler has been described previously including a video and outlined above [5]. The addition of the 10Fr redivac drain acts as a guide and safety mechanism for the insertion of the spindle of the AutoSuture CEEA™ circular stapler through the pursestring. The diameter of the CEEA™ stapler is also smaller, 28 or 31 mm, compared to the 33-mm EEA™ stapler, posing less of a risk of incorporating sphincter muscle into the stapler. We have reported on a series of 12 cases using the AutoSuture CEEA stapler in which there were no anastomotic leaks, and to date, all patients have had a good functional outcome [5]. A potential drawback of this technique is that it demands good visualisation of the pelvic floor and the rectal stump from the abdominal side before completing the anastomosis since the anvil is placed onto the stapling gun using conventional laparoscopic methods. In the difficult narrow pelvis with a short rectal stump, this exposure is sometimes limited. To overcome problems with abdominal exposure, whilst still avoiding the disadvantages of the wide 33-mm stapling device, a standard 28-mm stapler can be utilised using the pull-through method which relies on a good transanal view rather than abdominal. Further, it creates the possibility of a transanal anastomosis with excellent control of the distal pursestring. A potential disadvantage of this technique is the relative short anvil, which has to be clamped inside the anal canal in order to attach the stapler. Therefore, its use is not recommended in higher anastomoses above 4–5 cm. The author, Tuynman, who pioneered this technique has performed 36 cases so far and experienced two clinical leaks, both managed by transgluteal drain positioning.

The potential advantages and disadvantages of each anastomotic technique are outlined in Table 1. However, the true benefits and optimal approach are yet to be tested and confirm in comparative studies (Table 1).

**Table 1:** Comparison of hand-sewn and stapling techniques for coloanal and colorectal anastomoses post-transanal total mesorectal excision

Anastomotic technique	Advantages	Disadvantages
Hand-sewn coloanal	Suitable for coloanal and low colorectal anastomoses Suture placement and depth of suture controlled by surgeon under direct vision Avoids the difficult step of placing a rectal pursestring	Difficult anastomosis if a long rectal stump due to: Inadequate visual exposure Too far to reach with ‘open’ instruments Potentially worse functional outcomes compared to colorectal anastomoses
Stapled—EEA™ Haemorrhoid Stapler 33 mm	Long central rod allows passage through the anal canal and attachment to the spindle prior to pursestring closure Good for long rectal stumps	Large 33-mm stapler diameter posing a risk to adjacent structures, such as anal sphincters and vagina Needs sufficient rectal stump length to form the rectal pursestring
Abdominal double pursestring stapled—28- or 31-mm CEEA™ stapler	Smaller stapler diameter posing less risk to adjacent structures Precise placement of the anvil through the centre of the pursestring under direct vision Abdominal conventional anvil-stapling device attachment	Needs sufficient rectal stump length to form the rectal pursestring May be difficult to connect the anvil to the spindle laparoscopically in an obese narrow pelvis with poor visualisation
Transanal double pursestring stapled—28- or 31-mm CEEA™ stapler	Smaller stapler diameter posing less risk to adjacent structures Precise placement of the anvil through the centre of the pursestring under direct vision Transanal stapling technique for low anastomoses	Can be used only for low anastomoses. Good transanal exposure is essential and therefore not suitable for heights above 4 cm. For higher anastomoses, the two other techniques are preferred

Since each patient and each tumour has their own characteristics, it may be reasonable for a surgeon to be able to perform a number of anastomotic techniques in order to tailor the approach to the patient’s anatomy. This has been suggested in Knol et al.’s recent publication on technical aspects of TaTME, a more individualised approach may be better depending on the distance of the tumour from the anorectal junction (ARJ) [4]. This will determine whether a platform is used at the start of the transanal TME dissection and what the most favourable anastomotic technique will be. For example, see Table 2.

**Table 2:** Suggested cutoff distances of tumour from anorectal junction to determine the use of a platform to start the transanal dissection and subsequent anastomotic technique

Tumour distance from anorectal junction (cm)	Start of transanal TME dissection	Anastomotic technique
Coloanal	Without platform	Hand-sewn
2–3	With platform	28- or 31-mm CEEA™ stapler; transanal technique
3–4	With platform	28- or 31-mm CEEA™ stapler; abdominal technique
>4 or wide colon/pelvis	With platform	EEA™ Haemorrhoid Stapler

Regardless of the technique used, care should always be taken to ensure well-vascularised anastomotic ends, optimal visualisation, and awareness of the potential risk to nearby structures such as the anorectal sphincters and vagina, especially when adherent to the rectal wall.

Recently, Tuech et al. [8] published the first functional outcome results in 56 consecutive patients who underwent endoscopic transanal proctectomy (ETAP) and hand-sewn coloanal anastomosis for low rectal cancer. The overall morbidity after surgery was 26 % with three patients developing a clinical anastomotic leakage (none required reoperation) and a local recurrence rate of only 1.7 % (median follow-up: 29 months, range 18–52). It is reassuring to find that the median Wexner score after stoma reversal was 5 (range 3–18), and only three patients (5.7 %) required a colostomy due to severe faecal incontinence. Given the more distal tumours included in this study, all of which had hand-sewn coloanal anastomoses, functional results are likely to be even better following more proximal stapled anastomoses.

Two further groups have published their initial experience with TaTME including the Dutch group, Veltcamp Helbach et al. [9], and Dr Lacy [10] from Barcelona. Eighty patients underwent TaTME in the Dutch group [9]; stapled anastomosis using the EEA™ haemorrhoidal stapler was used in cases in which gastrointestinal continuity was restored. Post-operative complications were seen in 39 % of patients, nine of whom required reoperation. One patient returned to theatre due to anastomotic leak.

Lacy et al. [10] have published the largest case series of 140 patients to date. Hand-sewn coloanal anastomosis was performed for patients with the most distal rectal tumours, whilst for mid- and proximal tumours, an EEA 33-mm circular stapler was used. Major complications were seen in 10 % of cases, with anastomotic leaks detected in 12 patients (8.6 %), three treated successfully conservatively, whilst one required percutaneous drainage and two had rectal tube transanal and intravenous antibiotics. The remaining nine patients returned to theatre with one of these patients requiring a stoma. Anastomotic bleeding occurred in three patients of whom one underwent a reoperation for transanal reinforcing stitches to control the bleeding.

Studies specifically comparing hand-sewn versus stapled coloanal/colorectal anastomosis following TaTME have yet to be published. Similis et al. [11] conducted a systematic review including 37 studies with a total of 628 participants who underwent TaTME resection. The review found that 66 % of anastomoses were hand-sewn coloanal and only 34 % were stapled. Anastomotic leak occurred in 25 cases, anastomotic stenosis in 11, and fistula formation in one case. Due to the heterogeneity of the studies included, with a low number of stapled anastomoses and cases likely to have been performed at an early stage in the surgeon’s learning curve for TaTME, firm conclusions as to the optimal anastomotic method cannot be made. Anastomotic techniques have been compared following traditional laparoscopic and open rectal resections, with conflicting results. Cong et al. [12] found significantly lower rates of anastomotic leakage and stricture formation following stapled coloanal anastomosis compared to manual anastomosis following laparoscopic intersphincteric resections. The complication rates were similar for fistula formation, bleeding, and neorectal mucosal prolapse between the two groups. An earlier randomised study comparing hand-sewn versus stapled techniques in colonic J-Pouch-Anal anastomosis for rectal cancer found that anastomotic stricture rates were lower in the stapled group but did not reach statistical significance [13]. Post-operative morbidity and functional problems were similar between the two groups, but intra-operatively, the time taken to perform a stapled anastomosis was significantly faster. In 2012, a Cochrane review found insufficient evidence to demonstrate superiority of stapled over hand-sewn techniques in colorectal anastomosis surgery, regardless of the level of anastomosis [14]. The only statistically different results were that stricture formation was more frequent with stapling (*P* < 0.05), and the time taken to perform the anastomosis was longer with hand-sewn techniques.

As with all emerging techniques, small modifications and technical optimisation are often required to further enhance the feasibility and safety profile. Three anastomotic colorectal techniques post-TaTME are in practice, and this description allows tailoring of the technique to length of the anal canal and height of anastomosis. However, studies comparing these techniques with functional outcome have yet to be published. Ideally, large randomised studies are required to compare post-operative outcomes between hand-sewn and stapling groups. However, as stated by Professor Wexner, ‘the rapid adoption by inadequately trained low-volume surgeons may sadly jeopardize the ultimate achievement’ of TaTME. Therefore, structured training, skills acquisition, mentorship, and credentialing with a standardised surgical approach are essential requisites in order to elicit and achieve the true potential benefits of TaTME.

## Electronic supplementary material

Supplementary material 1 (MP4 199611 kb)

## References

[1] Atallah S, Albert M, Larach S (2010). Transanal minimally invasive surgery: a giant leap forward. Surg Endosc.

[2] Buess G, Theiss R, Hutterer F (1983). Transanal endoscopic surgery of the rectum—testing a new method in animal experiments. Leber Magen Darm.

[3] Rao P, Reddy N (2004) Per oral transgastric endoscopic appendectomy in human. In Proceedings of the 45th annual conference of the society of gastrointestinal endoscopy of India, Jaipur, pp 28–29

[4] Knol JJ, D’Hondt M, Souverijns G, Heald B, Vangertruyden G (2015). Transanal endoscopic total mesorectal excision: technical aspects of approaching the mesorectal plane from below—a preliminary report. Tech Coloproctol.

[5] Bracey E, Knol J, Buchs N (2015). Technique for a stapled anastomosis following transanal total mesorectal excision for rectal cancer. Colorectal Dis.

[6] Parks AG (1977). Endoanal technique of low colonic anastomosis. Surg Tech.

[7] Hompes R, Guy R, Jones O, Lindsey I, Mortensen N, Cunningham C (2014). Transanal total mesorectal excision with a side-to-end stapled anastomosis—a video vignette. Colorectal Dis.

[8] Tuech JJ, Karoui M, Lelong B (2015). A step toward notes total mesorectal excision for rectal cancer: endoscopic transanal proctectomy. Ann Surg.

[9] Veltcamp Helbach M, Deijen CL, Velthuis S, Bonjer HJ, Tuynman JB, Sietses C (2015) Transanal total mesorectal excision for rectal carcinoma: short-term outcomes and experience after 80 cases. Surg Endosc Apr 29. PMID: 25921202 10.1007/s00464-015-4221-y 25921202

[10] Lacy AM, Tasende MM, Delgado S (2015). Transanal total mesorectal excision for rectal cancer: outcomes after 140 patients. J Am Coll Surg.

[11] Simillis C, Hompes R, Penna M, Rasheed S, Tekkis PP (2015) A systematic review of transanal total mesorectal excision. Is this the future of colorectal surgery? Colorectal Dis. doi:10.1111/codi.13151 10.1111/codi.13151 26466751

[12] Cong JC, Chen CS, Ma AX, Xia ZX, Liu DS, Zhang FY (2014). Laparoscopic intersphincteric resection for low rectal cancer: comparison of stapled versus manual coloanal anastomosis. Colorectal Dis.

[13] Laurent A, Parc Y, McNamara D, Parc R, Tiret E (2005). Colonic J-pouch-anal anastomosis for rectal cancer: a prospective, randomized study comparing handsewn vs stapled anastomosis. Dis Colon Rectum.

[14] Neutzling CB, Lustosa SAS, Proenca IM, da Silva EMK, Matos D (2012). Stapled versus handsewn methods for colorectal anastomosis surgery. Cochrane Database Syst Rev.

